# Production efficiency differences between poikilotherms and homeotherms have little to do with metabolic rate

**DOI:** 10.1111/ele.13633

**Published:** 2020-11-09

**Authors:** Jaap van der Meer

**Affiliations:** ^1^ Wageningen Marine Research P.O. Box 57 Den Helder 1780 AB The Netherlands; ^2^ Department of Ecological Science Vrije Universiteit Amsterdam Amsterdam The Netherlands; ^3^ Department of Coastal Systems NIOZ Royal Netherlands Institute for Sea Research Den Burg The Netherlands

**Keywords:** Dynamic Energy Budget (DEB) theory, ecological efficiency, reproduction, somatic growth

## Abstract

The idea that homeothermic populations have a much lower production efficiency than poikilothermic populations, because warm‐blooded individuals exhibit a higher metabolic rate per gram of body weight, is widespread. Using Dynamic Energy Budget (DEB) theory, in combination with a modelling exercise based on empirical data for over 1000 different species, I show that this idea is wrong. Production efficiency of homeothermic individuals can be as high or even higher than that of poikilotherms. Differences observed are merely the result of different energy allocation and life‐history strategies. Birds, for example have evolved to invest a large proportion of the assimilated energy in somatic growth and maintenance and to mature at a relatively large size. Therefore, their production efficiency as an adult is low. This low reproduction efficiency combined with a low mortality rate causes the low production efficiency of bird (and other homeothermic) populations.

## Graphical abstract

The idea that homeothermic populations have a much lower production efficiency than poikilothermic populations, because warm‐blooded individuals exhibit a higher metabolic rate per gram of body weight, is widespread. Using Dynamic Energy Budget (DEB) theory, in combination with a modeling exercise based on empirical data for over 1000 different species, I show that this idea is wrong.

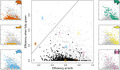

## INTRODUCTION

The ecological efficiency, also called Lindeman efficiency, is the ratio of the productivity of a trophic level and that of the level below (Lindeman, 1942). For an animal trophic level the ecological efficiency is the product of three types of efficiency: the consumption efficiency, which equals the ingested energy by a trophic level divided by the produced energy at the level below; the assimilation efficiency, which is the ratio of the assimilated energy and the ingested energy; and finally the production efficiency, which equals the ratio of the productivity of the trophic level and the assimilated energy (Reiss, 1989; Chapin *et al*., 2002).

Measuring these efficiencies in the field is not an easy task because a trophic level may consist of hundreds of different species, each with its specific efficiency. Studies of the energy budget of single populations may reveal generalities that could make it easier to collate the figures for all constituent populations within a trophic level. It has long been observed that the production efficiency of poikilothermic populations is much higher than that of homeothermic populations (Engelmann, 1966; Golley, 1968; McNeill and Lawton, 1970; Humphreys, 1979). Golley (1968), for example observed that about 20% of the assimilated energy by poikilothermic populations is manifested as production, whereas this figure is as low as 2% in homeothermic bird and mammal populations. He concluded that this difference should not come as a surprise as homeotherms exhibit a higher metabolic rate per gram of body weight than poikilotherms do. Lawton (1981) used the same argument and warned that basic physiological constraints should not be ignored when comparing efficiencies of endotherms and ectotherms. The idea that population efficiencies differ as a direct consequence of physiological differences among individuals has since then been repeated over and over again, not just in the ecological literature but also in the animal farming literature (Nakagaki and Defoliart, 1991; Torrissen *et al*., 2011; van Broekhoven *et al*., 2015; Fry *et al*., 2018). van Broekhoven *et al*. (2015) write that ‘Insects, being poikilotherms, do not use metabolic energy to maintain a constant body temperature as homeotherms do, and can therefore invest more energy into growth, resulting in a higher feed conversion efficiency'. The argument percolated into general ecological textbooks too, and Chapin *et al*. (2002), for example state that the high constant body temperature of homeotherms makes them inefficient in producing new animal biomass. The idea that poikilothermic individuals are much more efficient than homeothermic ones has been questioned by only a few authors (Wieser, 1984; Reiss, 1989), but they could not explain the results obtained for populations, such as, for example by Golley (1968).

Here, I explore what theoretical models of animal energy budgets predict about the production efficiency of individuals and populations. I start with three simple models, the well‐known Bertalanffy model (von Bertalanffy, 1934) and the two growth models of the Metabolic Theory of Ecology (West *et al*., 2001; Hou *et al*., 2008). Next, I examine the predictions of the standard Dynamic Energy Budget (DEB) model. DEB theory provides an integrated look at whole‐organism energetics and, in contrast to the simple models, includes reserve dynamics and reproduction. The standard DEB model has already been parametrised for over a thousand different species (Van der Meer *et al*., 2014; Marques *et al*., 2018) and the predicted efficiencies for all these species are calculated and presented. Most species examined are vertebrates. Eight example species are compared in some more detail. Five of these species are poikilotherms, three are homeothermic.

## Bertalanffy individuals and populations

Although von Bertalanffy (1934) thought of growth as a difference between anabolic and catabolic energy flows, the Bertalanffy growth equation can also be interpreted as the growth rate being the difference between food assimilation rate, which is surface‐area related, and maintenance rate, which is volume related (Paloheimo and Dickie, 1965). It can then be written in the form.

(1) $$\frac{dV}{dt}=\frac{aV^{2/3}‐bV}{q_{r}}$$

where V is the volume of the organism (length^3^), t is time, a is the area‐specific assimilation rate (energy length^−2^ time^−1^), b is the volume‐specific maintenance rate (energy length^−3^ time^−1^) and $q_{r}$ is the energy required to create one unit of volume (energy length^−3^). The production, expressed in energy, equals the energy content of the tissue created $q_{t}V$. As there are always overhead costs of growth, implying that the energy that is required for tissue growth is more than the chemical energy that the created tissue actually contains, it follows that $q_{t}<q_{r}$. The production rate $q_{t}dV/dt$ divided by the assimilation rate $aV^{2/3}$ thus equals.

(2) $$q\left(1‐\frac{b}{a}V^{1/3}\right)=q\left(1‐\frac{V^{1/3}}{V_{\infty }^{1/3}}\right)$$

where $q=q_{t}/q_{r}\;<\;1$. Hence, the production rate–assimilation rate ratio decreases linearly with length, starting at q when the organism is infinitely small and going to 0 when it reaches its ultimate size, which equals $V_{\infty }^{1/3}=a/b$. Indeed, when the organism's size is extremely small, the maintenance rate is negligible compared to the assimilation rate and all assimilated energy is put into growth, with a conversion efficiency of q. The organism approaches its ultimate size when the assimilated energy is just sufficient to pay for the maintenance, and no energy is left anymore for growth. Organisms with the same ultimate size do not differ in this relationship and the magnitude of the volume‐specific maintenance rate b, which may be much higher for homeothermic animals than for poikilothermic animals, is not relevant. Only the ratio b/a counts. The life‐time production‐assimilation ratio $r_{I}\left(t_{d}\right)$ for an individual that dies at age $t_{d}$ equals.

(3) $$r_{I}\left(t_{d}\right)=\frac{q_{t}V\left(t_{d}\right)}{\int_{0}^{t_{d}}aV{\left(t\right)}^{2/3}dt}$$

and this function too varies from close to q for animals that almost immediately die to almost 0 for those that become extremely old (Supplementary material).

For a stationary population the production‐assimilation ratio $r_{P}$ equals.

(4) $$r_{P}=\frac{\int_{0}^{\infty }f\left(x\right)q_{t}V\left(x\right)dx}{\int_{0}^{\infty }f\left(x\right)(\int_{0}^{x}aV{\left(y\right)}^{2/3}dy)dx}=\frac{q_{t}\int_{0}^{\infty }f(x)V(x)dx}{a\int_{0}^{\infty }S\left(x\right)V{\left(x\right)}^{2/3}dx}$$

where $f\left(x\right)$ is the density function of age at death x, $S\left(x\right)$ is the survival function and y is age. In case of constant instantaneous mortality rate equal to λ, the density function equals $f\left(x\right)=\lambda \;\exp\left(‐\lambda x\right)$ and the survival function $S\left(x\right)=\exp\left(‐\lambda x\right)$. Solving the integrals for this case (see, e.g. Van der Meer *et al.,* (2001)) reveals.

(5) $$r_{P}=q\frac{\lambda }{\lambda +b/q_{r}}$$

This result tells that the population production efficiency depends upon the volume‐specific maintenance rate b (scaled by the energy $q_{r}$ required to create it) relative to the instantaneous mortality rate λ. If the mortality rate is much higher than the scaled maintenance rate, the efficiency will approach the ratio q. This is logical, as a high mortality rate means that most animals die at a small size and these small animals have an efficiency, as was shown earlier, close to q. Homeothermic populations with their high maintenance rate can thus only be as efficient as poikilothermic populations when the mortality rate keeps pace with the maintenance rate. In reality, the contrary occurs, with longevity being high enough to approach maximum size and thus population production efficiency being low.

Analysis of the growth models of the Metabolic Theory of Ecology (West *et al*., 2001; Hou *et al*., 2008) reveals essentially the same result, but working with a scaling coefficient of 3/4 instead of 2/3 (Supplementary material).

## DEB modelling

The standard DEB model (Kooijman, 2010) organism has three succeeding life stages, the embryo, which neither feeds nor reproduces, the juvenile, which feeds but does not reproduce, and the adult, which feeds and reproduces. The organism is described by three state variables: (1) structural body volume V (length^−3^), (2) reserve density $\left[E\right]$ (energy length^−3^), which is the amount of reserves per unit of structural body volume and (3) maturity $E_{H}$ (energy), which is the cumulative energy allocated to development. The theoretical concept of reserves in the DEB model should not, as one may be inclined to do, be looked upon as material that is set aside for later use. DEB reserves should be considered as a central depot within individual cells, to which all assimilated material is dispatched and from where material is directly available for metabolic use. Maturity merely quantifies an amount of information and its energetic value is negligible. Hence, the cumulative energetic investment in maturity does not translate into an increase in energy content of the body, but dissipates as heat out of the body.

A list of assumptions gives rise to a set of coupled ordinary differential equations for the three state variables (see Kooijman (2010) for an extensive treatment of DEB theory, and for less detailed but gentler introductions Van der Meer (2006, 2016, 2019). Assumptions for the standard DEB model are, among other things, that (1) assimilation rate ${\dot{p}}_{A}$ is proportional to the surface area of the structural body, and at maximum food availability equals $\left\{{\dot{p}}_{\mathrm{Am}}\right\}V^{2/3}$, (2) all assimilated energy enters the reserves and is then mobilised from the reserves (the rate of changes in the reserves is thus the difference between the assimilation rate and the mobilisation rate), (3) a fixed fraction κ of the mobilisation rate is spent on maintenance, which is assumed proportional to structural body volume (it equals $\left[{\dot{p}}_{M}V\right]$), and on growth, assuming fixed costs $\left[E_{G}\right]$ for growth per unit volume, (4) embryos and juveniles develop, that is build up maturity, and for them the rate of change of maturity equals 1‐κ times the mobilisation rate minus the maturity maintenance costs, which are proportional to maturity, (5) transitions between embryo and juvenile (called birth) and between juvenile and adult (called puberty) occur at fixed levels of maturity, respectively, $E_{H}^{b}$ and $E_{H}^{p}$, (6) once the animal has become adult, it has reached its maximum maturity $E_{H}^{p}$ and starts to reproduce.

These assumptions result in the reserve density following first‐order dynamics and the rate at which the reserve density drops down in the absence of assimilation is proportional to the energy conductance parameter $\dot{v}$ and inversely proportional to structural body length $V^{1/3}$. The ratio between the area‐specific assimilation rate $\left\{{\dot{p}}_{\mathrm{Am}}\right\}$ and the energy conductance $\dot{v}$ gives the maximum reserve density.

At constant and maximum food availability, the DEB growth equation is given by.

(6) $$\frac{dV}{dt}=\frac{\kappa \left\{{\dot{p}}_{\mathrm{Am}}\right\}V^{2/3}‐\left[{\dot{p}}_{M}\right]V}{\kappa \left\{{\dot{p}}_{\mathrm{Am}}\right\}/\dot{v}+\left[E_{G}\right]}$$

Reserve density does not change at constant food availability, at least not when the mother has provided the embryo with the adequate amount of reserves. This implies that the structural volume V including the reserves $\left[E\right]V$, easily translates into energy content by the proportionally coefficient.

$$q_{t}=\frac{\left\{{\dot{p}}_{\mathrm{Am}}\right\}}{\dot{v}}+\left[E_{V}\right]$$

where $\left[E_{V}\right]$ is the energy density of the structural body. That is the energy content of the structural body and the reserves summed equals $E_{E+V}=q_{t}V$. See Table 1 for an explanation of all DEB parameters.

Hence at constant food conditions, the DEB model and the Bertalanffy model are very similar, as far as somatic growth and reserve dynamics are concerned. For the DEB model the somatic production rate (including reserves) $q_{t}dV/dt$ divided by the assimilation rate $\left\{{\dot{p}}_{\mathrm{Am}}\right\}V^{2/3}$ equals.

(7) $$\kappa q\left(1‐\frac{V^{1/3}}{V_{\infty }^{1/3}}\right)$$

where $V_{\infty }^{1/3}=\kappa \left\{{\dot{p}}_{\mathrm{Am}}\right\}/\left[{\dot{p}}_{M}\right]$. Hence the conclusions of the previous section on production efficiency more or less hold for the standard DEB model, apart of course from the reproduction part, which will be discussed next. Table 2 provides the DEB interpretation of the Bertalanffy parameters.

The rate of change of maturity equals 1‐κ times the mobilisation rate minus the maturity maintenance costs, which are proportional to maturity. Hence.

(8) $$\frac{dE_{H}}{dt}=\left(1‐\kappa \right){\dot{p}}_{C}‐{\dot{k}}_{J}E_{H}$$

for $E_{H}\;<\;E_{H}^{p}$. When the animals have become mature (at puberty), that is $E_{H}=E_{H}^{p}$, maturity does not change anymore and $dE_{H}/dt=0$ and the energy is channelled into reproductive material. The rate at which the energy in reproductive material builds up is.

(9) $$\frac{dE_{R}}{dt}=\kappa_{R}\left(\left(1‐\kappa \right){\dot{p}}_{C}‐{\dot{k}}_{J}E_{H}^{p}\right)$$

where $\kappa_{R}$ is the efficiency at which invested energy in reproduction is actually stored in reproductive material. This parameter is supposed to be close to one, as, for example no major biochemical transformations are involved in transferring adult reserves into juvenile reserves within the egg. It can be shown that the mobilisation rate ${\dot{p}}_{C}$ equals.

$${\dot{p}}_{C}=\frac{\left\{{\dot{p}}_{\mathrm{Am}}\right\}/\dot{v}}{\kappa \left\{{\dot{p}}_{\mathrm{Am}}\right\}/\dot{v}+\left[E_{G}\right]}\left(\dot{v}\left[E_{G}\right]V^{2/3}+\left[{\dot{p}}_{M}\right]V\right)$$

The contribution of reproduction to the production efficiency is indicated by the ratio between the reproduction rate, that is the rate at which energy is stored in reproductive material d $E_{R}$/d t, and the assimilation rate ${\dot{p}}_{A}$. Unfortunately, this ratio is thus given by a complicated expression. For fully grown adults (for which $V=V_{\infty }$ and ${\dot{p}}_{C}=\left\{{\dot{p}}_{\mathrm{Am}}\right\}V_{\infty }^{2/3}$) the ratio simplifies to.

(10) $$\kappa_{R}\left(\left(1‐\kappa \right)‐\frac{{\dot{k}}_{J}E_{H}^{p}}{\left\{{\dot{p}}_{\mathrm{Am}}\right\}V_{\infty }^{2/3}}\right)$$

For illustrative reasons I can make one extra assumption that simplifies this expression even further. In DEB theory, the ratio $\left[{\dot{p}}_{M}\left[/\right]E_{G}\right]$ is called the ‘maintenance rate coefficient’ ${\dot{k}}_{M}$. It stands for the maintenance costs of structure relative to the investment, and the reader might recall the similar ratio $b/q_{r}$, that popped up earlier in the analysis of the Bertalanffy model. When ${\dot{k}}_{J}={\dot{k}}_{M}$, which means that the relative maintenance costs of maturity equal those of the somatic body, it can be shown (Kooijman, 2010) that.

$$E_{H}=\frac{1‐\kappa }{\kappa }\left[E_{G}\right]V$$

Hence setting ${\dot{k}}_{J}={\dot{k}}_{M}$ implies that maturity, which is reached when $E_{H}=E_{H}^{p}$, occurs at a fixed volume $V_{p}$. One consequence is that for fully grown animals, equation 9 simplifies to.

(11) $$\frac{dE_{R}}{dt}=\kappa_{R}\left(1‐\kappa \right)\left(\left\{{\dot{p}}_{\mathrm{Am}}\right\}V_{\infty }^{2/3}‐\frac{\left[{\dot{p}}_{M}\right]}{\kappa }V_{p}\right)$$

and the reproduction rate–assimilation rate ratio simplifies to

(12) $$\kappa_{R}\left(1‐\kappa \right)\left(1‐\frac{V_{p}}{V_{\infty }}\right)$$

Clearly, this ratio is, apart from the reproduction efficiency parameter $\kappa_{R}$, only determined by two dimensionless (compound) parameters, the fraction of the mobilised energy spend on somatic growth and maintenance κ and the ratio between the volume at puberty and the ultimate volume. Possible differences in this ratio between homeothermic and poikilothermic individuals thus bear no direct relationship with a higher maintenance rate of homeotherms.

## Fitting the standard DEB model

At present, the Dynamic Energy Budget model has been fitted for more than two thousand species. The results were downloaded from the add‐my‐pet website at https://www.bio.vu.nl/thb/deb/deblab/add_my_pet/index.html on February 12, 2020. All 1329 species that were fitted with the standard model (the so‐called std and stx models) were selected. This selection contained 420 mammals, 451 birds, 161 reptiles, 112 amphibians, 138 other chordates, most of them either ray‐finned fishes (78) or cartilaginous fishes (49), and finally 47 invertebrates.

For eight species some more detailed results are presented as examples. Four of these species, the water flea *Daphnia magna*, the Antarctic krill *Euphausia superba*, the western fence lizard *Sceloporus occidentalis*, and the black‐capped chickadee *Poecile atricapillus* were selected because their fits were based on extensive data sets, containing food intake rates, growth rates and reproduction rates. The other four species, two homeotherms and two poikilotherms, were, mainly for illustrative reasons, taken from various outer regions of the space spanned by the relevant DEB parameters (such as κ), which implies that these species show different energy allocation strategies. These species are the thicklip grey mullet *Chelon labrosus*, the Bengal monitor *Varanus bengalensis*, the black bear *Ursus americanus* and Peters' dwarf epauletted fruit bat *Micropteropus pusillus*.

**Figure 1:**
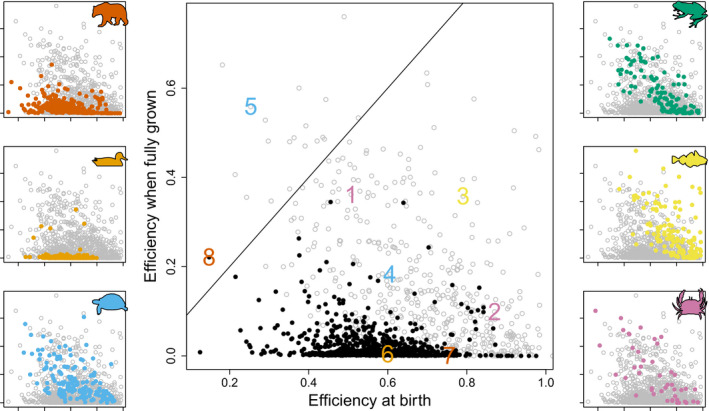
Production efficiency of fully grown adults, given by the ratio between reproductive rate and assimilation rate, vs. the production efficiency at birth, given by the ratio between growth rate and assimilation rate, for 1392 species. In the main central panel mammals and birds are shown by black solid circles and all other groups by grey open circles. The side panels show the same points, but coloured for a specific group, on the left side mammals, birds and reptiles, on the right side amphibians, other chordates and other phyla. Numbers in the main panel refer to the eight example species, see Table 3 for explanation. The diagonal line gives all points where the two efficiencies are equal.

All classes (mammals, birds, reptiles, birds, ray‐finned fishes and cartilaginous fishes) show a very large variation in the production efficiency at birth, ranging, for example for reptiles from 0.24 to 0.93, for mammals from 0.13 to 0.98 and for birds from 0.26 to 0.80 (Fig. 1). The variation in the production efficiency of fully grown animals is lower, especially for birds (Fig. 1). The production efficiency of fully grown reptiles ranges from almost zero to 0.61, and 95% of the birds have an efficiency as an adult lower than 0.015.

**Figure 2:**
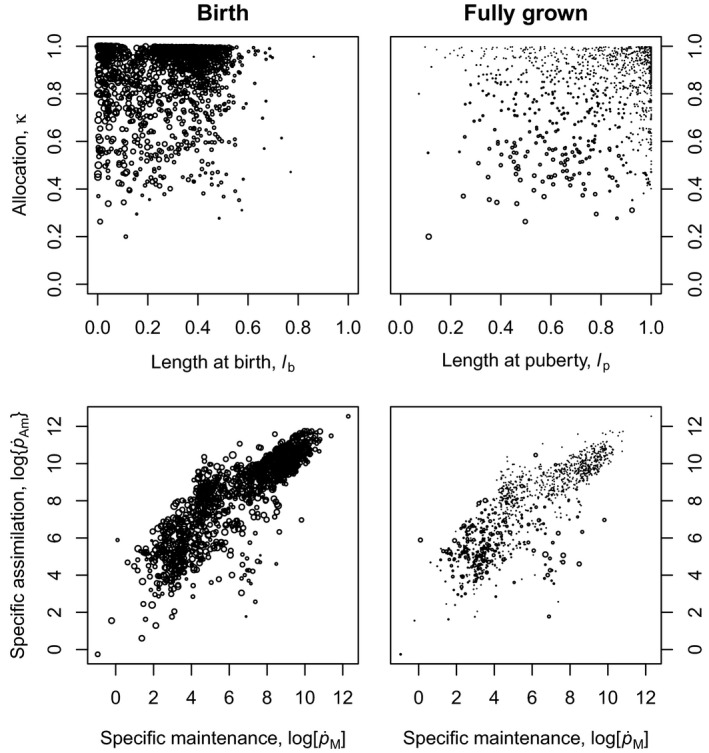
The DEB parameter κ, which is the fraction of the mobilisation rate spent on somatic maintenance and growth, vs. relative length at birth, that is when feeding starts (upper left panel) and at puberty, that is when reproduction starts (upper right panel). The DEB parameter $\left\{{\dot{p}}_{\mathrm{Am}}\right\}$, which is the area‐specific assimilation rate, vs. $\left[{\dot{p}}_{M}\right]$, which is the volume‐specific maintenance rate (lower panels). The size of the circles is proportional to the production efficiency at birth (left panels) or when fully grown (right panels).

**Figure 3:**
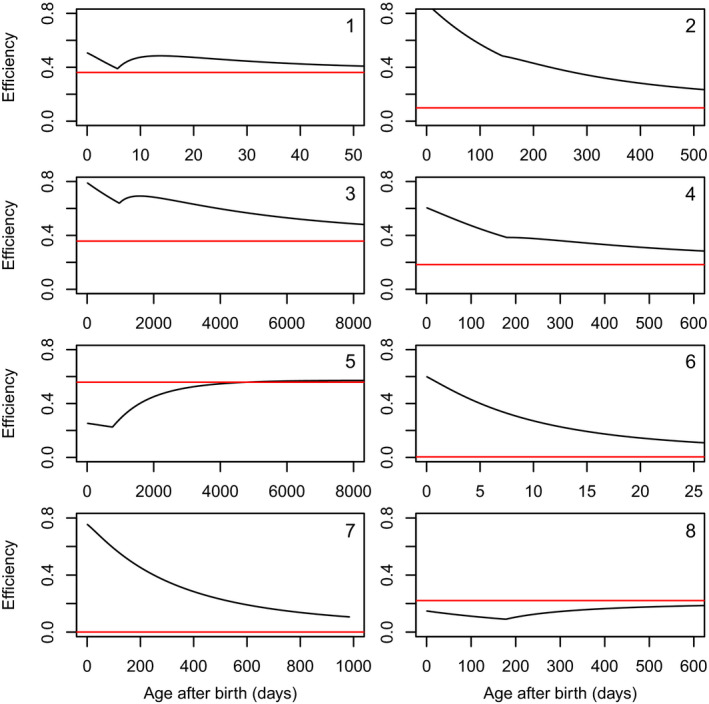
Life‐time production efficiency vs. age after birth. The efficiency is given by the ratio between the energetic content of the body including reserves plus the cumulative energetic investment in reproduction from puberty to the age indicated (numerator) and the cumulative assimilation rate from birth to the age indicated (denominator). Eight selected species, see Table 3. The discontinuity occurs at the age of puberty, see text for further explanation.

Theory predicted that the efficiency at birth is mainly governed by κ and by the relative length at birth $l_{b}=V_{b}^{1/3}/V_{\infty }^{1/3}$ (eqn 7), which is confirmed by the model fits (Fig. 2). The higher κ and the lower $l_{b}$, the higher the production efficiency at birth. Antarctic krill and the thicklip grey mullet combine a high allocation fraction to soma with a very low relative size at birth and indeed have a high production efficiency in early life (Figs 1 and 3, Table 3). This also holds for the black bear, who is born at a very small size relative to the size of its parents. The black bear is born with a weight of less than 300 g, whereas its father can weigh as much as 130 kg. The opposite is true for the fruit bat, who allocate a large fraction of the mobilised energy to reproduction and whose birth size (15.5 g) is very large compared to the size of its mother (35 g).

Theory also predicted that the efficiency of fully grown individuals is governed by κ and by the relative length at puberty (eqns 10 and 11). But now the rules are: The lower κ and the lower $l_{p}$, the higher the production efficiency as a fully grown adult (Fig. 2). Two examples: the black‐capped chickadee has, as most other birds, a very high allocation fraction to soma plus a high relative size at puberty, which causes a very low production efficiency later in life as an adult. In contrast, the bengal monitor has a very low allocation fraction to soma and reaches puberty at a relatively small size, and therefore even shows a higher production efficiency as adult than as newly born juvenile (Figs 1 and 3, Table 3).

The DEB parameters $\left[{\dot{p}}_{M}\right]$ and $\left\{{\dot{p}}_{\mathrm{Am}}\right\}$ determine the size‐specific maintenance and assimilation rates and indirectly the metabolic rate. A consistent pattern between these parameters and production efficiency could not be found (Fig. 2).

## Discussion

The idea that homeothermic populations have a much lower production efficiency than poikilothermic populations because homeothermic individuals exhibit a higher metabolic rate per gram of body weight than poikilothermic individuals do, has been firmly settled in the minds of many ecologists and livestock scientist, see, for example Chapin *et al*. (2002). If one compares two organisms of the same size and with the same assimilation rate, the efficiency of the poikilotherm will indeed be much higher, as it spends much less on maintenance and heating. It is tempting to conclude from this observation that production efficiency has to be less in homeotherms vs. poikilotherms. But if one describes this comparison in terms of, for example the Bertalanffy model as given above, one will see that such a conclusion is not warranted. Assume equal size ($V_{1}^{*}=V_{2}^{*}=V^{*}$), equal specific assimilation rate ($a_{1}=a_{2}=a$), equal energy density and overhead costs of growth ($q_{1}=q_{2}=q$), but much lower maintenance costs for the poikilotherm ($b_{1}<<b_{2}$), where the index ‘1’ refers to the poikilotherm and ‘2’ to the homeotherm. The efficiency, given by $q\left(1‐\left(b_{i}/a\right)V^{*}\right)$ (eqn 2), is thus indeed much higher for the poikilotherm. But this comparison is only a particular snapshot during their lives. The poikilotherm in the example will grow to a much larger size (maximum length is given by $a/b_{i}$), so the comparison is in fact between a ‘small' poikilotherm (relative to its ultimate size) and a ‘large' homeotherm. This is the main underlying reason that the poikilotherm has a higher efficiency in the presented example. If two species of the same size that will also reach the same ultimate size are compared, their efficiency is the same, because the ratio a/b is similar. The higher specific maintenance rate of the homeotherm is then associated with a higher specific assimilation rate.

The Bertalanffy model further predicts that animals that reach the same ultimate size (and thus have the same ratio a/b) can only differ in their growth efficiency due to a difference in the parameter q. This parameter is given by the ratio of the energy content of the tissue created ($q_{t}$) and the energy required to create a unit of tissue ($q_{r}$, which is roughly the sum of overhead cost and the energy content of the tissue). These parameters are generally believed to be rather similar across species, which would imply that there will not be much difference in production efficiency in the case of the same maximum body size. The DEB model prediction is slightly more complicated, as the allocation parameter κ and the maximum reserve density ($\left\{{\dot{p}}_{\mathrm{Am}}\right\}/\dot{v}$) also play a role. The more is allocated to growth and somatic maintenance and the more reserves there are, the higher the growth efficiency is. The variability in these parameters might also explain the results of a recent study that suggests that overhead costs of growth can be very low and are much more variable than previously thought (Ferral *et al*., 2020). Here there is room for further explorations. There are, however, no reasons to believe that these differences in allocation, reserve density and overhead costs of growth have anything to do with the homeotherm‐poikilotherm dichotomy.

Within the standard DEB model an extra area‐specific heating term $\left\{{\dot{p}}_{T}\right\}V^{2/3}$ could have been included, where the parameter $\left\{{\dot{p}}_{T}\right\}$ stands for the area‐specific heating, and such term can also easily be added to the Bertalanffy model (e.g. $hV^{2/3}$). This would have changed the efficiency predictions. In this case, the growth efficiency of the Bertalanffy model as given in eqn 2 is multiplied by a factor $\left(a‐h\right)/a$. The term $V_{\infty }$ in the simplified reproduction rate–assimilation rate ratio of the DEB model (eqn 12) changes into $\left(V_{\infty }^{1/3}+\left\{{\dot{p}}_{T}\right\}/\left[{\dot{p}}_{M}\right]\right)V_{\infty }^{2/3}$. However, for none of the mammals and birds there was a need to include such extra surface‐area‐related heating term in the DEB model fitting procedure. Apparently, the high volume‐related maintenance costs, which include activity, etc., substitute for all thermoregulation costs (Humphries and Careau, 2011). The underlying idea that animals spend most of their time in their thermoneutral zone (with respect to the field metabolic rate) is also confirmed by the fact that in many birds and mammals even basal metabolic rate hardly differs between summer and winter (McKechnie et al., 2015; Schaeffer *et al*., 2020). When differences are observed they rarely exceed more than 30%, whereas a factor of 10‐15 would be required if the productivity of mammalian or avian populations were to be brought into line with the productivity of individual mammals or birds.

Predictions made with the theoretical standard DEB model, which was fitted to empirical data for more than a thousand species, confirmed that the production efficiency of growing birds and mammals can be just as high or even higher than that of growing reptiles, amphibians or fishes. This is exemplified by newly born black bears who have a much higher production efficiency than newly hatched Bengal monitors. The production efficiency of adult birds is indeed much lower than that of, for example adult water fleas, but that is the result of a different energy allocation strategy and not of a higher avian metabolic rate. Birds have evolved to invest a high proportion of the mobilised energy in somatic growth and maintenance (they have a large value for κ) and to mature at a relatively large size, and therefore their production efficiency as an adult is low. This, in combination with a relatively low mortality rate in comparison to a high physiological rate, causes the low production efficiency of bird populations (and other homeothermic populations) compared to poikilothermic populations.

The temperature‐dependent assimilation and maintenance rate parameters (which affect metabolic rate) did not show a clear relationship with production efficiencies. This should not come as a surprise as the relevant equations for the production efficiencies (eqns 7, 10 and 11) do not contain these parameters. Besides, these equations only contain ratios of rates, and temperature effects will thus always cancel out.

Although the on average low production efficiency of fully grown mammals and birds is directly linked to a specific energy allocation and life‐history strategy (i.e. high fraction towards soma and large relative size at puberty), one might wonder whether unknown physiological constraints exist that have forced homeotherms to adopt such strategy. It is therefore interesting to explore whether artificial selection could or already has resulted in, for example adult birds with a much higher production efficiency. It has been mentioned above that 95% of the birds in the add‐my‐pet data collection have, when fully grown, a production efficiency lower than 0.015. But there are a few bird species with much higher efficiency, as can be seen in Fig. 1. The collection basically contains only wild animals, but with the exception of a few husbandry and poultry species. One of these is the white leghorn *Gallus gallus domesticus*. This chicken lays on average 0.82 egg per day resulting in a production efficiency equal to 0.34. Such efficiency is comparable to that of the water flea and the thicklip grey mullet and much higher than the western fence lizard achieves. It is, of course, also much higher than that of the wild jungle fowl *Gallus gallus* that lays six eggs a year and has a production efficiency as an adult as low as 0.007.

This study used DEB theory, which provides an integrated look at whole‐organism energetics. By recognising that animals only function as a whole individual, this study was able to falsify the hitherto prevailing notion that metabolic rate alone can explain the difference in production efficiency among animal groups.

## AUTHORSHIP

Single author paper.

**Table 1:** Primary parameters of the standard DEB model

Symbol	Dimension	Interpretation	Process
$\left\{{\dot{p}}_{\mathrm{Am}}\right\}$	$eL^{‐2}t^{‐1}$	Surface‐area‐specific maximum assimilation rate	Assimilation
$\kappa_{X}$	–	Digestion efficiency	Digestion
$\dot{v}$	$Lt^{‐1}$	Energy conductance	Mobilisation
κ	–	Fraction of mobilisation rate spent on maintenance plus growth	Allocation
$\left[{\dot{p}}_{M}\right]$	$eL^{‐3}t^{‐1}$	Volume‐specific maintenance rate	Turnover/activity
$\left[E_{G}\right]$	$eL^{‐3}$	Volume‐specific costs of growth	Growth
${\dot{k}}_{J}$	t‐1	Specific maturity maintenance	Regulation/defence
$\kappa_{R}$	–	Reproduction efficiency	Egg formation
$E_{H}^{b}$	e	Maturity at birth	Life history
$E_{H}^{p}$	e	Maturity at puberty	Life history

**Table 2:** The DEB interpretation of the Bertalanffy model

DEB process or parameter name	Bertalanffy	DEB
Assimilation rate	$aV^{2/3}$	$\left\{{\dot{p}}_{\mathrm{Am}}\right\}V^{2/3}$
Area‐specific assimilation rate channelled to soma	a	$\kappa \left\{{\dot{p}}_{\mathrm{Am}}\right\}$
Volume‐specific maintenance rate	b	$\left[{\dot{p}}_{M}\right]$
Volume‐specific costs of growth including reserves for growth	$q_{r}$	$\kappa \left\{{\dot{p}}_{\mathrm{Am}}\right\}/\dot{v}+\left[E_{G}\right]$
Volume‐specific energy content of structure including reserves	$q_{t}$	$\left\{{\dot{p}}_{\mathrm{Am}}\right\}/\dot{v}+\left[E_{V}\right]$

**Table 3:** Estimates of the DEB parameters κ, $\left\{{\dot{p}}_{\mathrm{Am}}\right\}$ and $\left[{\dot{p}}_{M}\right]$ and the DEB predictions for the relative lengths at birth $l_{b}$ and at puberty $l_{p}$ for eight example species. The dimensionless parameter κ is the fraction of the mobilisation rate spent on somatic maintenance and growth. The parameters $\left\{{\dot{p}}_{\mathrm{Am}}\right\}$, which is the area‐specific assimilation rate and $\left[{\dot{p}}_{M}\right]$, which is the volume‐specific maintenance rate, are given at the body temperature T

Number	Species	κ ‐	$\left\{{\dot{p}}_{\mathrm{Am}}\right\}$ Jd^−1^ cm^−2^	$\left[{\dot{p}}_{M}\right]$ Jd^−1^ cm^−3^	$l_{b}$ ‐	$l_{p}$ ‐	T °C
1	Water flea	0.581	313	1200	0.170	0.457	20
2	Antarctic krill	0.895	238	214	0.030	0.586	9
3	Thicklip grey mullet	0.619	247	12	0.004	0.256	15
4	Western fence lizard	0.786	1047	612	0.355	0.709	24
5	Bengal monitor	0.356	350	6.7	0.207	0.353	25
6	Black‐capped chickadee	0.995	16568	10074	0.359	0.951	42
7	Black bear	0.927	7465	194	0.138	0.998	37
8	Peters' dwarf epauletted fruit bat	0.472	1171	234	0.770	0.917	35

### Peer Review

The peer review history for this article is available at https://publons.com/publon/10.1111/ele.13633.

## Supporting information

Supplementary Material

## Data Availability

No original data have been used.
