# Supplementary material for: Production efficiency differences between poikilotherms and homeotherms have little to do with metabolic rate
Source: Ecol Lett. 2020 Nov 9;24(2):219–26. doi: 10.1111/ele.13633 (PMC7821020; doi:10.1111/ele.13633)
Supplement: Supplementary file 1 — Supplementary Material [file ELE-24-219-s001.pdf]

Supplementary Material to Production efficiency differences between poikilotherms and homeotherms have little to do with metabolic rate

Jaap van der Meer[1,2,3]

[1]Wageningen Marine Research, P.O. Box 57, 1780 AB Den Helder, The Netherlands, jaap.vandermeer@wur.nl, +31 317 488105

[2]Vrije Universiteit Amsterdam, Department of Ecological Science, Amsterdam, The Netherlands

[3]NIOZ Royal Netherlands Institute for Sea Research, Department of Coastal Systems, Den Burg, The Netherlands

October 28, 2020

1 Supplementary material

1.1 Life-time efficiency

The Bertalanffy model as presented in the main text predicts that the production of an animal that lives up to age t_d equals $q_t L_\infty^3 (1 - e^{-kt_d})^3$, where the ultimate length $L_\infty = a/b$, and the Bertalanffy coefficient $k = b/3q_r$. Life-time assimilation is given by

$$aL_\infty^2 \int_0^{t_d} (1 - e^{-kt})^2 dt \tag{1}$$

After integration of this latter term, the following production-assimilation ratio results

$$\frac{q_t L_\infty (1 - e^{-kt_d})^3}{a(t_d - \frac{2}{k}(1 - e^{-kt_d}) + \frac{1}{2k}(1 - e^{-2kt_d}))} \tag{2}$$

1.2 MTE's ontogenetic growth models

The growth models of the Metabolic Theory of Ecology use a similar model as Von Bertalanffy's for growth, i.e.

$$\frac{\mathrm{d}m}{\mathrm{d}t} = \frac{B_0 m^{3/4} - B_m m}{E_m} \tag{3}$$

The original ontogenetic growth model (OGM) uses $B_0 m^{3/4}$ as the supply term (West et al., 2001) and efficiencies can be derived as before. The extended ontogenic growth model (EOGM) of the Metabolic Theory of Ecol-

ogy (Hou et al., 2008) takes a different direction and uses

$$(f + \gamma)B_0 m^{3/4} - \gamma B_m m \tag{4}$$

as the assimilation term. The ratio between the production rate and the assimilation rate is therefore given by

$$\frac{q_t}{E_m} \frac{B_0 m^{3/4} - B_m m}{(f + \gamma)B_0 m^{3/4} - \gamma B_m m} \tag{5}$$

The two models are explained in detail in van der Meer (2019).

References

Hou, C., Zuo, W., Moses, M. E., Woodruff, W. H., Brown, J. H., and West, G. B. (2008). Energy uptake and allocation during ontogeny. *SCIENCE*, 322(5902):736–739.

van der Meer, J. (2019). Metabolic theories in ecology: The Dynamic Energy Budget theory and the Metabolic Theory of Ecology. In Fath, B., editor, *Encyclopedia of Ecology (Second Edition)*, volume 3, pages 463–471. Elsevier, second edition.

West, G. B., Brown, J. H., and Enquist, B. J. (2001). A general model for ontogenetic growth. *NATURE*, 413:628–631.
